# Harnessing antiviral RNAi therapeutics for pandemic viruses: SARS-CoV-2 and HIV

**DOI:** 10.1007/s13346-025-01788-x

**Published:** 2025-01-20

**Authors:** Ellen Bowden-Reid, Ernest Moles, Anthony Kelleher, Chantelle Ahlenstiel

**Affiliations:** 1 The Kirby Institute, UNSW Sydney, Sydney, 2052 Australia; 2 Children’s Cancer Institute, Lowy Cancer Research Centre, UNSW Sydney, Sydney, 2052 Australia; 3 Australian Centre for Nanomedicine, Faculty of Engineering, UNSW Sydney, Sydney, 2052 Australia; 4 School of Clinical Medicine, Medicine and Health, UNSW Sydney, Sydney, 2052 Australia; 5 UNSW RNA Institute, UNSW Sydney, Sydney, 2052 Australia

## Abstract

Using the knowledge from decades of research into RNA-based therapies, the COVID-19 pandemic response saw the rapid design, testing and production of the first ever mRNA vaccines approved for human use in the clinic. This breakthrough has been a significant milestone for RNA therapeutics and vaccines, driving an exponential growth of research into the field. The development of novel RNA therapeutics targeting high-threat pathogens, that pose a substantial risk to global health, could transform the future of health delivery. In this review, we provide a detailed overview of the two RNA interference (RNAi) pathways and how antiviral RNAi therapies can be used to treat acute or chronic diseases caused by the pandemic viruses SARS-CoV-2 and HIV, respectively. We also provide insights into short-interfering RNA (siRNA) delivery systems, with a focus on how lipid nanoparticles can be functionalized to achieve targeted delivery to specific sites of disease. This review will provide the current developments of SARS-CoV-2 and HIV targeted siRNAs, highlighting strategies to advance the progression of antiviral siRNA along the clinical development pathway.

**Graphical Abstract:**

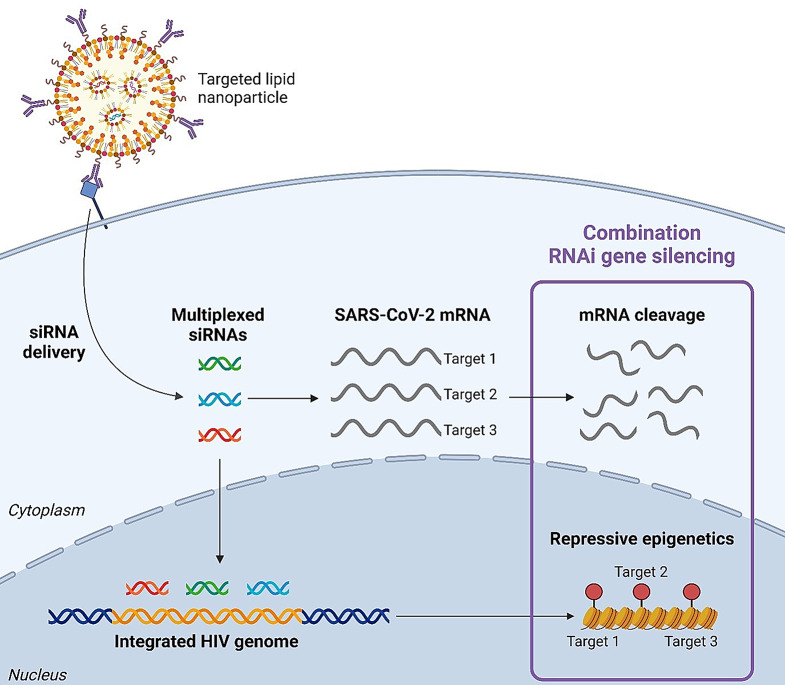

## RNAi therapeutics for pandemic viruses: a turning point

Many viral infections were declared among some of the significant threats to global health by the World Health Organization (WHO) in 2019 [1]. Amongst this list, Human Immunodeficiency Virus (HIV), Dengue, Ebola and other high-threat pathogens including Zika, Nipah, Middle East Respiratory Syndrome coronavirus (MERS-CoV) and Severe Acute Respiratory Syndrome (SARS) were implicated as posing a significant risk. The imminent threat of Influenza or pathogen X (representative of an unknown pathogen) and a country’s health emergency preparedness and response systems were also acknowledged as key determinants of future public health [1]. The rapid emergence of SARS-CoV-2, which was categorized as a pandemic in March 2020 [2], exposed the vulnerability of global health systems in the face of novel pathogens. In response, the world saw the swift, record-breaking development of the first ever mRNA vaccines [3, 4] which are estimated to have contributed to saving tens of millions of lives in the first year alone [5].

The rapid development and approval of Pfizer-BioNTech’s BNT162b2 [4] and Moderna’s mRNA-1273 [3] SARS-CoV-2 vaccines was significant for many reasons. They highlighted the value of innovative technologies, such as RNA-based approaches, when it comes to tackling infectious diseases that threaten the global community. In addition, it demonstrated the potential versatility and agility of RNA-based therapeutics and vaccines, and how they can be rapidly adapted for clinical needs. Importantly, they provided evidence of safety and efficacy of mRNA technologies and their delivery systems in a global population, as well as scalability of manufacturing, logistics and storage. This propelled the field of RNA-based approaches into the mainstream, which has engaged the pharmaceutical industry, investors, government and public, and become a pivotal turning point for the field. This momentum will be crucial for the future development of novel RNA strategies in the prevention, treatment, and control of other infectious diseases.

Broadly speaking, an RNA therapeutic encompasses a wider group of drugs that use an RNA based molecule to selectively alter the expression of genes or gene products [6]. RNA interference (RNAi) therapies are a class of therapeutics within this group with shared clinical success alongside the mRNA vaccines. However, despite having the potential to revolutionize the clinical approach to many acute and chronic viral infections, the full capabilities of RNAi therapeutics are yet to be harnessed. As the SARS-CoV-2 pandemic finishes its fifth year, and the HIV epidemic enters its fifth decade, the impact novel RNAi therapeutic solutions could bring cannot be overstated. In this review, we provide new perspectives by comparing both the classical RNAi pathway (post-transcriptional gene silencing; mRNA cleavage) as well as the novel RNAi pathway (transcriptional gene silencing; epigenetic regulation), which is often overlooked in short-interfering (siRNA) therapeutics development. In fact, all siRNA therapeutics approved by the United States Food and Drug Administration (FDA) utilize the classical RNAi pathway. This allows us to highlight the importance of how applying both pathways can achieve far-reaching clinical applicability for both acute and chronic virus infections, exemplified here by COVID-19 and HIV, respectively. Additionally, we will discuss current platforms for siRNA transport and intracellular delivery in vivo, with a focus on lipid-based nanoparticles (LNPs), as well as targeting systems used to achieve site-specific delivery.

## The RNAi pathways

RNAi, which was first described in 1998 [7], refers to the evolutionarily conserved mechanism of gene regulation, where a small non-coding RNA ‘interferes’ with a target gene. The concept of harnessing RNAi to treat disease gained significant momentum when Andrew Fire and Craig Mello received the joint Nobel Prize in Physiology or Medicine in 2006 for their work describing this phenomenon, and coining the phrase “RNA interference” [7, 8]. In their study, they discovered that injecting exogenous double-stranded RNA (dsRNA) into the nematode worm *Caenorhabditis elegans*, silenced the specific gene that the dsRNA was patterned on, with an accompanied elimination of the corresponding mRNA. Soon thereafter, it was demonstrated that the dsRNA mediated silencing by inducing sequence specific degradation of mRNA [9]. This phenomenon had indeed already been reported in the literature years prior to Fire and Mello in petunias [10, 11] and fungi [12], however the mechanisms underlying the authors’ observations were unknown at the time. In parallel, a second distinct pathway of RNAi was emerging. Initially described in transformed tobacco plants [13], and later discovered to be conserved within humans [14], this arm of RNAi was found to regulate gene expression via the epigenetic inactivation of a target gene promoter.

**Fig. 1:**
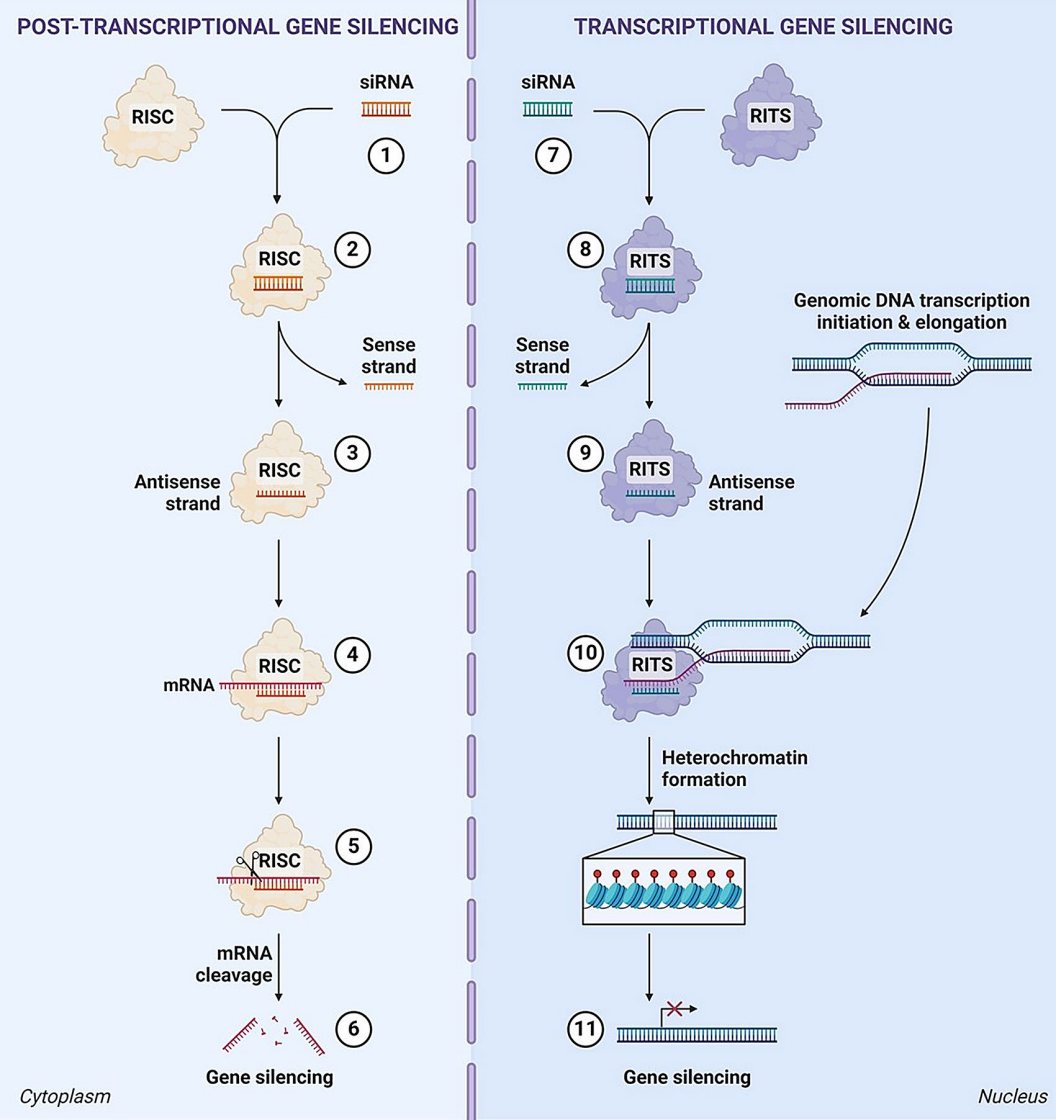
RNA interference pathways induced by synthetic siRNA. 1) An siRNA designed to a target mRNA enters the cytoplasm and 2) complexes with the inactive multiprotein RNA-induced silencing complex (RISC). 3) The sense strand of the siRNA duplex is removed, leaving the antisense strand complexed in the now active RISC. 4) The RISC is guided by the antisense strand to its target mRNA where it binds via homologous base pairing, 5) initiating endonucleolytic cleavage of the target mRNA. 6) Translation of the target mRNA is inhibited, and post-transcriptional gene silencing is achieved. 7) An siRNA designed to the promoter of a target gene enters the nucleus and 8) complexes with the inactive multiprotein RNA-induced transcriptional silencing complex (RITS). 9) The sense strand of the siRNA duplex is removed, leaving the antisense strand complexed in the now active RITS. 10) The RITS is guided to its target loci and binds to promoter associated transcripts via homologous base pairing. 11) Upon binding, histone methyltransferases are recruited to induce heterochromatin formation and nascent transcript degradation resulting in transcriptional gene silencing of the target gene locus. Created with BioRender.com

Both arms of RNAi are now known to be induced by short-interfering RNA (siRNA) (~ 21 bp) [15, 16], micro-RNA (miRNA) (~ 22 bp) [17, 18] and P element-induced wimpy testis (PIWI)-associated RNA (~ 26–31 bp) [19, 20], with the former two having been more extensively characterized. An siRNA functions to downregulate gene expression in a sequence specific manner and will typically only function on a single target [21]. In contrast, most miRNA will contain multiple mismatches to their target mRNA sequence and can act by regulating up to hundreds of genes simultaneously [22]. While several therapeutic miRNAs are in development [23], the high potential of causing unforeseen off-target toxicities due to the broad mechanism of action of miRNAs remains a key hinderance to their clinical progression. One notable example is the termination of a Phase I clinical trial of MRX34, *i.e.*, an miRNA mimetic to treat advanced solid tumors, due to serious immune-mediated adverse events that led to the death of four participants [24]. As such, only siRNAs have progressed to human use in the clinic [25–30]. The cytosolic and nuclear pathways of RNAi are known as post-transcriptional gene silencing (PTGS) and transcriptional gene silencing (TGS), respectively (Fig. 1). When an exogenous siRNA enters the cytoplasm, the duplex interacts with Argonaute 2 (Ago2) protein [31], containing catalytic activity [32], of the inactive RNA-induced silencing complex (RISC) [33]. To become active, the RISC must undergo a maturation step that involves separating the two strands of the siRNA and discarding the sense strand, while the antisense strand is left in complex with the RISC [31, 34]. The mature RISC is subsequently guided to a target mRNA homologous to the sequence of the antisense strand [33]. Through homologous base pairing, the antisense strand and mRNA bind, catalyzing the endonucleolytic cleavage of the target mRNA by Ago2 and consequently preventing its translation into protein [32]. Through the potent, but transient (in the range of days), suppression of viral mRNA products, therapeutic siRNA offer an alternative class of antivirals for acute viral infections such as SARS-CoV-2, Respiratory syncytial virus (RSV) and Ebola virus. Currently, all FDA approved siRNA therapeutics utilize PTGS to treat their target diseases, which are discussed in the section “RNAi therapeutics”.

TGS is the lesser known and less well understood nuclear pathway of RNAi, which uses a promoter-targeted siRNA to execute its gene silencing function. Following entry into the nucleus, an exogenous siRNA duplex interacts with Argonaute 1 (Ago1) protein [35] of the RNA-induced transcriptional gene silencing complex (RITS) [36, 37]. Like PTGS, activation of the RITS occurs when the sense strand is removed from the complex, however how this occurs in humans is still unknown. Through sequence complementarity, the RITS is guided to the target loci, where it binds to promoter associated transcripts via base pairing [38, 39]. Binding induces the degradation of the nascent transcript and the recruitment of histone methyltransferases that epigenetically modify the site of the promoter [16]. Other repressive epigenetic markers that are reported to be enriched during TGS include histone deacetylase 1 (HDAC1) [38], with a reduction in histone acetylation also reported [40]. These modifications initiate and maintain heritable heterochromatin formation [41] of the siRNA target site, resulting in longer-term gene silencing in the range of weeks [38]. While the TGS mechanism and protein components of RITS have been described in yeast and plants [36], the mode of action and protein components is less clear in humans [36]. This is due to the lack of human gene homologs for several of the known yeast RITS protein components. However, there are unrelated human genes that encode proteins with similar domain structures and function, suggesting that the human RITS may function similarly to lower eukaryotes. As such, partly due to the unknown nature of human RITS, the development of TGS-inducing therapeutic siRNA are still at the proof-of-concept stage. Nevertheless, they possess enormous potential for treating chronic virus infections such as HIV, Human papilloma virus (HPV), Hepatitis B virus (HBV), Epstein-Barr virus (EBV), Herpes simplex virus (HSV), Varicella zoster virus (VZV) and Cytomegalovirus (CMV).

### RNAi therapeutics

As of December 2024, six siRNA have received FDA approval to treat human diseases. The approval of the first siRNA drug [25] was no easy feat– 30 years following the discovery of RNAi, this breakthrough cost an accumulation of 16 years of research and an estimated $2.5 billion of investment by Alnylam Pharmaceuticals.

In 2018, the FDA approved the first RNAi drug Patisiran [25]. Patisiran uses siRNA to inhibit abnormal transthyretin (TTR) production, caused by a mutation in the *TTR* gene, to treat the resulting disease hereditary transthyretin-mediated amyloidosis (hATTR). Since this exciting milestone, an additional five therapeutic siRNA have received FDA approval for various genetic diseases. Givosiran (approved 2019) treats acute hepatic porphyria by suppressing the over-activation of the delta-aminolevulinate synthase 1 enzyme, which results in the toxic accumulation of porphyrin molecules [26]. Lumasiran (approved 2020), a therapy developed for primary hyperoxaluria type 1 where excessive amounts of oxalate are produced, was the first siRNA to indirectly treat the disease by targeting a gene upstream of oxalate production [27]. Inclisiran, which received approval in 2021, is a low-density lipoprotein (LDL)-cholesterol lowering therapy for atherosclerotic cardiovascular disease [28]. It functions by inhibiting proprotein convertase subtilisin/kexin type 9, which would normally increase levels of circulating LDL-cholesterol. In 2022, the FDA approved Vutrisiran, a next generation hATTR treatment with enhanced uptake, shortening the dosing requirement from once every 3 weeks with Patisiran to once a quarter with Vutrisiran [29]. Most recently, Nedosiran received approval in September 2023 to also treat primary hyperoxaluria type 1 [30]. Like Lumasiran, Nedosiran indirectly reduces oxalate overproduction, but does so by targeting a different enzyme that catalyzes oxalate formation. Patisiran, Givosiran, Lumasiran, Inclisiran, Vutrisiran and Nedosiran have a shared commonality; they all treat diseases that manifest in the liver, where the disease-causing mRNA is predominantly produced. It is likely that the clinical success of these therapies is highly dependent on their hepatic targets, via simplified drug delivery, and is discussed in further detail in the section “Nanoparticle-based siRNA delivery systems in the clinic”.

In addition to hereditary conditions, siRNAs have long been explored as antiviral therapies in preclinical and clinical research. In fact, the first in human proof-of-concept studies of siRNA were performed by Alnylam using an siRNA targeting RSV [42, 43]. Although the RSV targeted siRNA failed to meet all secondary endpoints establishing antiviral efficacy in this and subsequent trials [44, 45], these studies still highlighted the potential for siRNA as antiviral therapeutics. More recently, therapeutic siRNA for chronic HBV infection, also a liver manifesting disease, has seen the most success to date, with the results of a Phase I clinical trial recently released [46]. This early exploratory trial of the N-acetyl-D-galactosamine (GalNAc)-conjugated siRNA, administered by subcutaneous injection, found the siRNA to be safe and well tolerated. This type of siRNA modification is introduced separately in the section “Nanoparticle-based siRNA delivery systems in the clinic”. In addition, siRNA treatment resulted in marked reductions of Hepatitis B surface antigen, maintained for over a year in individuals previously treated with nucleos(t)ide analogue therapy [46]. A Phase II trial exploring whether other strategies are required to improve the effectiveness of this functional cure (sustained Hepatitis B surface antigen loss for six months following treatment discontinuation) are currently underway (ClinicalTrials.gov ID NCT04225715). Other novel siRNA therapeutics for chronic HBV have also progressed to Phase II trials, with results pending (NCT04856085, NCT04412863, NCT06154278). In addition to chronic HBV, which lacks curative therapies, many acute and chronic viral infections lack specific, potent treatment options, and would benefit from the clinical translation of novel therapeutics, such as siRNAs, highlighting two virus-specific examples using SARS-CoV-2 and HIV.

**Fig. 2:**
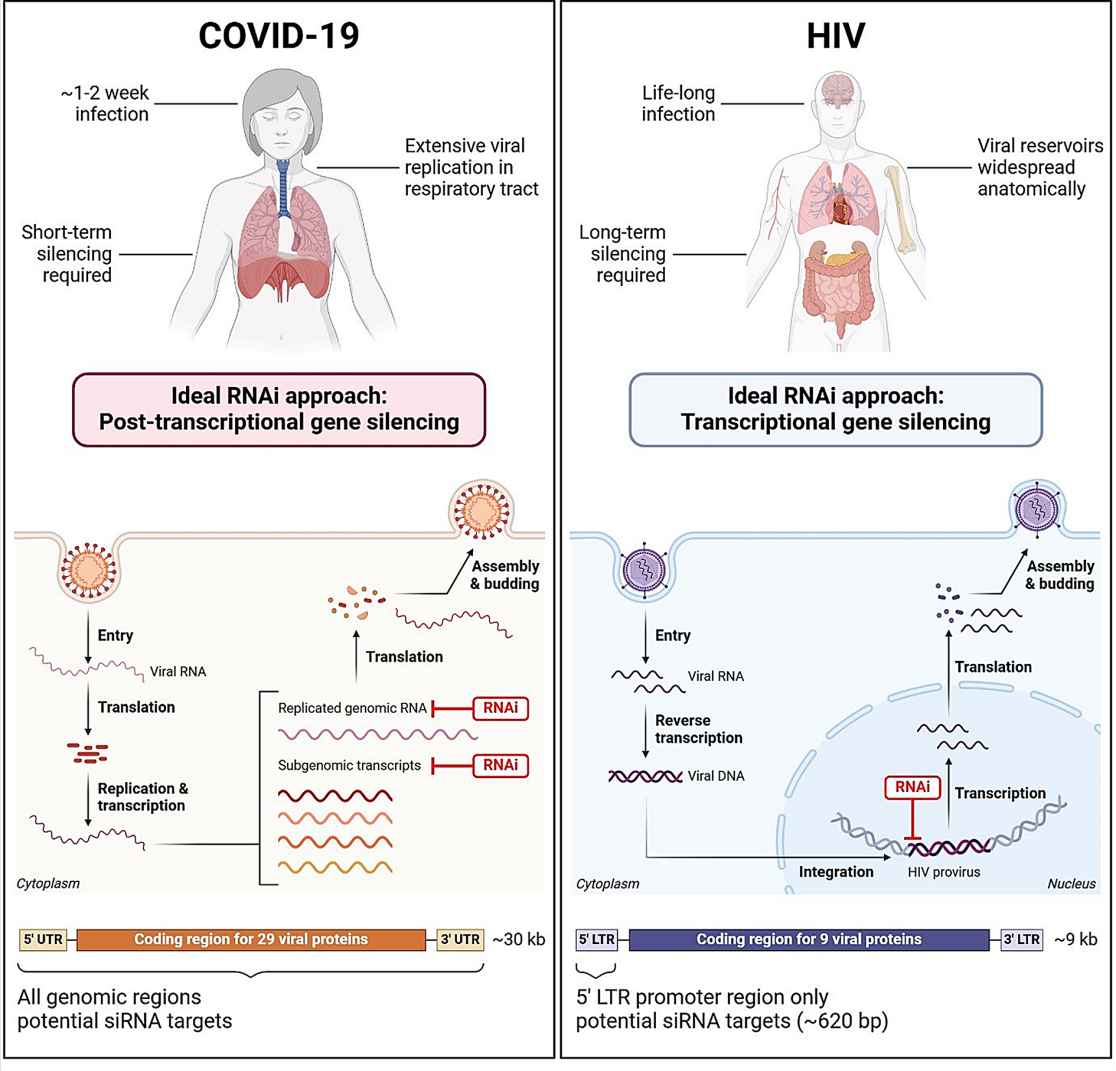
Differences between COVID-19 and HIV influencing RNAi approach and siRNA design strategy. Created with BioRender.com

SARS-CoV-2 is an acute infection, typically lasting 1–2 weeks, which involves extensive viral replication in the respiratory tract [47]. As such, viral suppression via a therapeutic is only required short-term. Viral replication occurs in the cytoplasm and involves the generation of subgenomic transcripts and genomic RNA [47], which are all possible targets of RNAi via PTGS. In comparison, HIV is a life-long infection with viral reservoirs widespread anatomically [48]. Treatment is required to induce sustained viral suppression, ideally with minimal doses. Viral replication occurs in the cytoplasm as well as the nucleus, where it is permanently integrated into the host cell genome. This integrated form of HIV, known as the provirus, serves as the template for the transcription of all viral mRNAs [48]. Thus, the promoter of the provirus represents the ideal target of RNAi via TGS, where the promoter can be deactivated via repressive epigenetics. The key differences between SARS-CoV-2 and HIV disease, life cycles and genomes that influence the RNAi approach and siRNA design are depicted in Fig. 2. Both of these diseases will be discussed in further detail in the sections below.

## RNAi therapies for acute infections: a look at COVID-19

Four years following the emergence of SARS-CoV-2, there have been almost 800 million cases and over 7 million reported deaths worldwide [49], however the true figures are estimated to be much higher [50]. Most cases of COVID-19 in vaccinated or pre-exposed individuals result in mild illness requiring no treatment, however severe and life-threatening disease can occur, with a striking 22% of the global population at risk of developing severe COVID-19 [51]. Although vaccination efforts have been incredibly effective at reducing morbidity and mortality associated with COVID-19, older adults and individuals who are immunocompromised/immunosuppressed or have comorbidities remain vulnerable to increased infection and mortality rates, as SARS-CoV-2 continues to circulate globally. Despite this, there is still a limited armamentarium of effective SARS-CoV-2 specific antivirals, even as we enter the sixth year of the pandemic.

The rapid evolution and continuing emergence of variants of concern have challenged the development of novel antiviral therapies, especially monoclonal antibodies (mAbs). Treatment of COVID-19 predominantly focuses on symptom management and a limited number of antivirals exist for people requiring therapeutic intervention. To date, four drugs have received FDA approval for the treatment of COVID-19. Remdesivir, initially developed to treat Hepatitis C virus, was the first repurposed antiviral to receive approval [52], but intravenous (IV) administration has limited its widespread utility. To overcome this, oral derivatives have since been developed, with the results of Phase III trials now released [53, 54]. Baricitinib [55] and Tocilizumab [56], both immunomodulatory drugs developed for a variety of inflammatory conditions such as rheumatoid arthritis, were next to receive approval, although they are reserved for certain hospitalized adults only [55, 56]. Most recently, Paxlovid, a combination pill containing the HIV protease inhibitor Ritonavir and pan-SARS protease inhibitor Nirmatrelvir, became the first oral antiviral pill to be approved by the FDA for COVID-19 treatment [57]. However, Ritonavir, which is included as a boosting agent to increase the half-life of Nirmatrelvir, restricts the use of Paxlovid, due to significant drug-drug interactions primarily caused by the Ritonavir component [57]. Many mAbs have also received Emergency Use Authorization (EUA) by the FDA throughout the course of the pandemic. While being specific for SARS-CoV-2, spike mutational escape, that is driven by antibody-based selection pressures, ultimately renders them ineffective with the emergence of new variants. Even mAb cocktails, such as Evusheld, Ronapreve and Bamlanivimab/Etesevimab, are no longer EUA approved due to the high prevalence of non-susceptible variants [58–60]. Additionally, all current antivirals must be administered within a 5–7 day window of symptom onset, further limiting their use [52, 57, 61].

While repurposing clinically approved drugs is certainly appealing to expedite many of the regulatory hurdles involved in drug development, there remains a lack of potent and therapeutically selective treatment options for SARS-CoV-2. While the number of cases and deaths appear to be dramatically declining, the reported figures do not accurately reflect actual infection rates due to the worldwide reduction in testing and reporting. There is clearly still an unmet need to continue developing treatment options with direct-acting antiviral activity against SARS-CoV-2, including antivirals that are resistant to mutational escape and ideally broad-spectrum in order to combat virus evolution of the entire SARS-coronavirus family. This, in combination with frequent vaccination, will be crucial for longer-term sustained disease prevention, control and management.

### SARS-CoV-2 targeted siRNA

Highly specific suppression of SARS-CoV-2 can be achieved through the use of RNAi. Each of the genome regions of SARS-CoV-2, which include four structural proteins (spike, envelope, nucleocapsid and membrane), 16 non-structural proteins and additional accessory proteins [62], are all druggable targets using siRNA. In addition, being able to target non-spike regions of the virus is particularly advantageous for avoiding immune-driven mutational escape in future variants. In addition, therapeutic siRNA can be designed to target sequences conserved across SARS-CoV-2 and SARS-CoV-1, which share 80% sequence similarity overall [63] and share regions of at least 19 bp that are 100% homologous. Indeed, we designed a panel of siRNAs that target highly conserved regions between both human and animal SARS-CoV-1 and SARS-CoV-2 against seven viral regions [64], which is discussed in further detail below. This strategic design against other betacoronaviruses will be important to ensure prospective broad-spectrum coverage and pandemic preparedness against future zoonotic spillover events.

Using antiviral siRNA therapeutics for coronaviruses was explored after the outbreak of SARS-CoV-1 in 2003, with initial success being observed both in vitro [65–67] and in Rhesus macaques in vivo [68]. Similarly, efforts into developing SARS-CoV-2 targeted siRNA began following the emergence of COVID-19 (summarized in Table 1).

We have previously demonstrated that siRNAs, with highly conserved target sites between human and animal SARS-CoV-1 and SARS-CoV-2, are effective against multiple lineages of SARS-CoV-2 ranging from the ancestral strain through to Omicron variants in vitro [64]. These antiviral siRNAs significantly reduced viral nucleocapsid mRNA levels by up to 99.9% and virus-induced cell death by up to 97% in live virus assays [64]. We also demonstrated that multiplexing two siRNAs, to simulaneously target combinations of non-structural protein 1, membrane and/or nucleocapsid mRNAs, increased antiviral potency and broad-spectrum capabilities, likely to withstand viral evolution [64]. Smaller studies by Tolksdorf et al. validated an siRNA target sequence, conserved between SARS-CoV-1 and SARS-CoV-2, to be efficacious against both viruses in vitro, with marked reductions in viral replication and viral cytopathic effect inhibition [69, 70]. A polymer-based nanoparticle was also demonstrated to deliver antiviral siRNA with reductions of SARS-CoV-2 genomic RNA of up to 75% in vitro, in air-liquid interface and 3D tissue culture systems [71, 72]. While biodistribution studies in mice showed uptake of the siRNA in lung epithelial cells with minimal inflammatory response, the authors did not test for efficacy against SARS-CoV-2 infection in vivo [71].

The potential of siRNAs for COVID-19 has been demonstrated in other in vivo studies. Idris et al. showed that IV administration of an siRNA encapsulated in an LNP at time of infection, followed by two re-doses, repressed SARS-CoV-2 in the lungs with a delayed onset of symptoms in infected mice, conferring a survival advantage [73]. The authors further developed their LNP formulation for intranasal (IN) delivery for viral elimination in the lung. When administered prophylactically, a significant reduction in SARS-CoV-2, measured using a viral immunoplaque assay, was observed in the lungs, however the antiviral efficacy was more than 50-fold lower when compared to IV delivery [74]. In a recent study, Idris et al. opted to re-design their siRNA targets in SARS-CoV-2, while maintaining their LNP formulation, due to the lack of potency in the nasal cavity [75], highlighting the importance of designing effective and highly potent siRNA targets. In a study using a SARS-CoV-2 RNA-dependent RNA polymerase targeted siRNA, Chang et al. demonstrated that aerosol inhalation or IN delivery in vivo in mouse models could effectively deliver a single, naked siRNA (with modifications to increase stability), inhibiting virus production and reducing lung pathology associated with COVID-19 disease [76]. The results of a Phase I randomized, double-blind placebo-controlled clinical trial administering this therapeutic via inhalation are pending (NCT05677893), however a Phase II study has been designed and is now recruiting (NCT05941793).

Khaitov et al. complexed their SARS-CoV-2 RNA-dependent RNA polymerase targeted siRNA with a biocompatible cationic peptide dendrimer (exact formulation undisclosed due to intellectual property) for delivery. When delivered daily for up to 6 days via aerosol administration in vivo, Syrian hamsters were observed to have a dose-dependent reduction in viral titres and decrease in disease pathology in the lung [77]. In a Phase II open-label clinical trial, this same drug was administered twice a day via inhalation for 14 days in hospitalized patients with moderate COVID-19. The authors found their drug to be safe and well tolerated as well as reducing recovery time compared to the control group receiving standard recommended therapies [78]. A Phase IIb-III clinical trial has since been completed and is awaiting report (NCT05783206).

Developing a broad-spectrum SARS-CoV-2 therapeutic will be critical for the ongoing management of cases of COVID-19. The studies highlighted here have each utilized similar siRNA design strategies, with therapeutic siRNA targeting conserved, and thus likely evolutionarily advantageous, regions of the SARS-CoV-2 or SARS-CoV-1 and SARS-CoV-2 genomes. In addition, where performed, subsequent conservation analyses found these target sites to remain highly conserved in evolved variants that emerged following initial design. Despite this, the identification of antiviral siRNAs still required screening large panels of siRNAs, even in studies where additional siRNA design tools were employed, such as siDESIGN Center (Horizon Discovery) [69, 70] and siSPOTR [71, 72, 79], to increase the likelihood of designing functional siRNAs.

With regards to siRNA chemical design, it is important to consider the inclusion of chemical modifications, such as 2’-OMe and phosphorothioate linkages, which increase siRNA stability whilst lowering immunogenicity. Notably, reducing immunogenicity is key to effectively administer siRNA therapeutics to cells of the respiratory tract. However, it is important to note that chemical modifications can also reduce antiviral potency compared to their unmodified counterparts [70, 73], thus these should be appropriately screened case by case. An additional challenge with respiratory administration is preserving the structural integrity of siRNA delivery vehicles following aerosolization. Indeed, several studies identified effective delivery vehicles for administration through the respiratory tract, including LNPs [73–75], naked but chemically modified siRNA [76] and a peptide dendrimer [77, 78], which could withstand aerosolization and/or nebulization. The latter two, which has progressed beyond Phase I clinical trials, emphasizes the clinical potential of utilising siRNAs for SARS-CoV-2.

However, due to the sequence specificity of RNAi, and the propensity of an siRNA to drive mutational escape, the use of a single siRNA for SARS-CoV-2 RNAi therapies in clinical trials raises concerns regarding the development of drug resistance and ongoing breadth of protection. A more pragmatic approach is a therapeutic containing multiple siRNAs, that target different viral regions simultaneously to develop a robust antiviral as we have previously proposed [64]. Surprisingly, multiplexing siRNAs for SARS-CoV-2 was explored by only a few studies, and therefore there is an urgent need to expand in this area to reach antiviral success in the long-term. In addition, multiplexing siRNAs offers the opportunity to develop a pan-SARS-coronavirus therapeutic, targeting highly conserved viral regions, with effectiveness across SARS-CoV-1, SARS-CoV-2 and possible emerging SARS-coronaviruses. Establishing such therapeutic portfolios for the next coronavirus or viral threat is essential for epidemic and pandemic preparedness strategies. However, while designing siRNAs with an inherent cross-virus approach can be achieved using highly conserved genomic targets, it should be noted that further work is still required to enable faster screening and identification of therapeutic candidates in rapid response to emerging novel viruses. Nevertheless, SARS-CoV-2 represents an ideal candidate for RNAi therapy to meet the urgent need for new therapeutic options.

**Table 1:** Summary of SARS-CoV-2 targeted siRNA studies

SARS-CoV-2 target(s)	Development stage	Type of studies	Delivery vehicle	Ref
M, N, NSP1	Preclinical	In vitro	Lipofectamine	[64]
5’UTR	Preclinical	In vitro	Lipofectamine	[69, 70]
NSP2, NSP9	Preclinical	In vivo	VIPER polyplex	[71, 72]
5’UTR, Helicase, RdRp	Preclinical	In vivo	LNP	[73, 74]
RdRp	Clinical	Phase II	Naked (modified)	[76]
RdRp	Clinical	Phase III	Peptide dendrimer	[77, 78]

Abbreviations: LNP, lipid nanoparticle; M, Membrane; NSP1, Non-structural Protein 1; NSP2, Non-structural Protein 2; NSP9, Non-structural Protein 9; N, Nucleocapsid; RdRp, RNA-dependent RNA-polymerase; UTR, untranslated region; VIPER, virus-inspired polymer for endosomal release

## RNAi therapies for chronic disease: the ongoing pursuit of an HIV cure

The first official reports of acquired immune deficiency syndrome (AIDS) were published in the early 1980s [80]. In the decades since, the prevalence of HIV has certainly grown to pandemic proportions, with WHO estimating that up to 113 million people have lived with HIV, and up to 51 million people have died of AIDS-related deaths [81]. The development of antiretroviral therapy (ART) has transformed this once (almost) guaranteed fatal infection into a chronic, manageable disease for people with HIV on treatment [82]. The most commonly used ART regimes consist of three drugs, from at least two different classes, used in combination [82]. While pharmaceutical companies have produced several well tolerated, easy to take, fixed-dose single pill ART options, a person’s control of HIV is dependent on life-long treatment, which is complicated by possible side effects, drug interactions, compliance issues, stigma and cost to the individual and healthcare systems.

Despite the tremendous strides that have been made in prevention and treatment of HIV, globally, there are approximately 39 million people currently living with HIV [83]. Annually, 1.3 million new infections and over 600,000 deaths from HIV-related causes still occur, disproportionally affecting low to middle income countries and vulnerable populations that are often marginalized, stigmatized and criminalized [81, 83]. As such, key components of the global HIV research agenda have highlighted the necessity of continued efforts in prevention and vaccination, as well as the development of an HIV cure [84]. Success of such ambitions will likely require investment into innovative technologies, such as RNA therapeutics.

### The HIV latent reservoir– challenges to a cure

A critical element of the HIV life cycle (like any retrovirus) is the reverse transcription of the single-stranded RNA genome into double-stranded proviral DNA, which is permanently inserted into the host cell chromosomal DNA [85]. This provirus (*i.e.*, the integrated DNA form of HIV) is the template for all viral mRNAs, whose transcription is driven by the 5’ long terminal repeat (LTR), which functions as the viral transcriptional promoter [86–88]. Integration of the HIV genome into the host genome also allows the virus to persist for as long as the host cell survives.

During primary infection, a viral reservoir of HIV is established predominantly in CD4^+^ T cells [89–91]. Within this reservoir, a proportion of cells harbor transcriptionally silent, or latent, provirus that is genetically intact and replication competent, capable of reactivating to produce infectious virus. Persistence of the reservoir is driven by immune evasion, clonal expansion and homeostatic proliferation [92, 93]. Due to this, ART treatment is required to be life-long [89–91]. As there is a lack of proof-reading during the HIV replication cycle, the reservoir is also comprised of cells containing defective provirus. While this does not contribute to the persistence of the reservoir via the production of new replication competent virus, some defective genomes can produce viral proteins that are immunogenic and contribute to persistent inflammation [94].

Following integration, epigenetic mechanisms that promote the heterochromatization of the nucleosomes associated with the 5’LTR promoter have been found to contribute to establishing and maintaining HIV latency (reviewed in [95]). In latently infected cells, several transcriptional repressors that recruit histone deacetylases (HDACs) have been shown to associate with the 5’LTR [96–99]. Increased methylation of histone 3 lysine 9 (H3K9) [100, 101] and histone 3 lysine 27 (H3K27) [102] via histone methyltransferases (HMT), supportive of silenced gene expression, also correlate with viral latency. In addition to histone repressive marks, DNA methylation of the cytosine-phosphate-guanidine (CpG) rich regions in the HIV promoter, which can recruit the binding of transcriptional repressors or inhibits the binding of transcriptional activators, are key drivers of latency [103, 104].

**Fig. 3:**
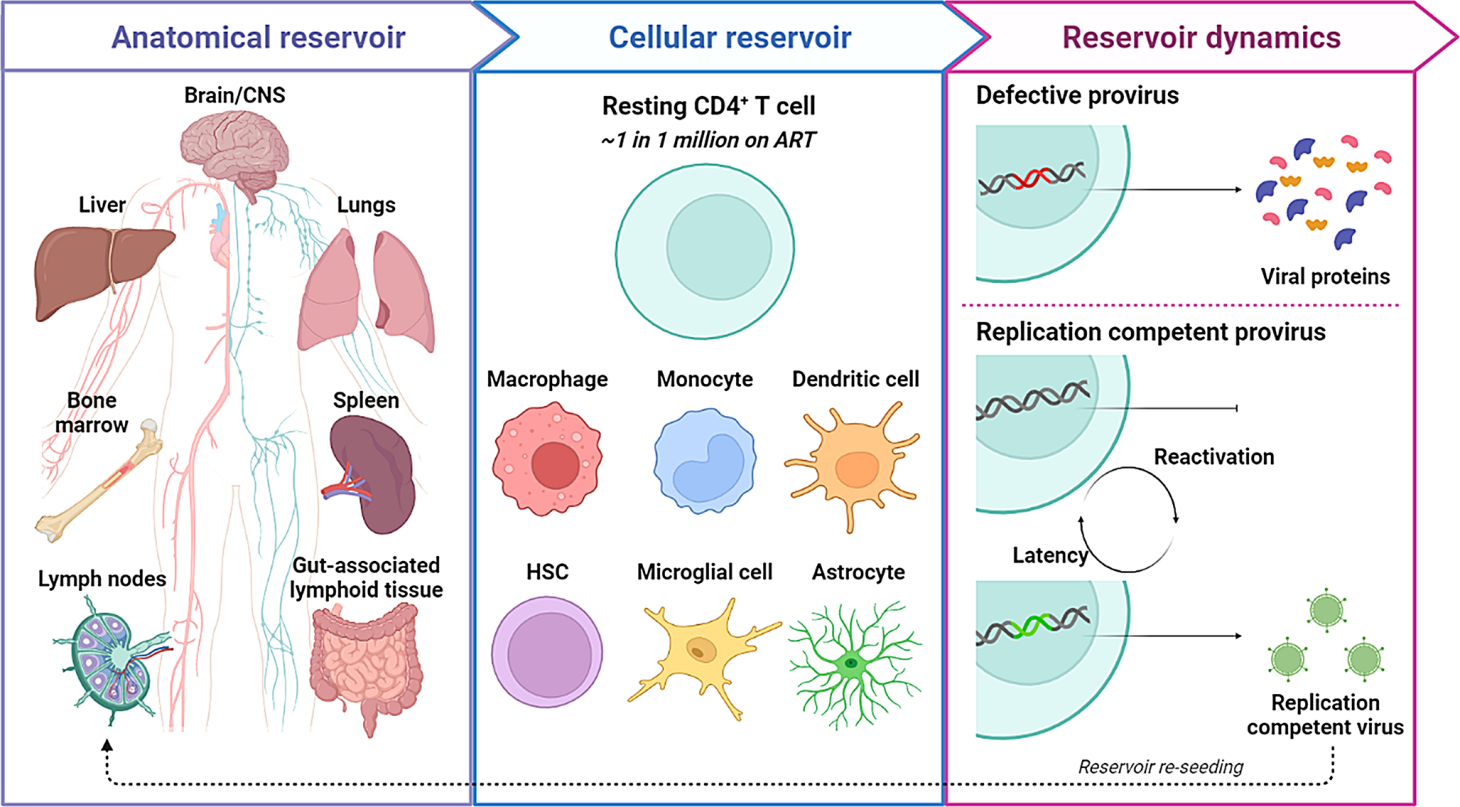
Challenges to achieving an HIV cure. Cells harboring latent HIV are anatomically widespread and have been found in the brain/central nervous system, lungs, spleen, gut-associated lymphoid tissue, bone marrow, liver and lymph nodes and in circulation in the lymphatic and cardiovascular systems. Resting memory CD4^+^ T cells are the primary reservoir of latent HIV, with approximately one in one million cells harboring latent virus in individuals on prolonged ART. Other cells implicated in the persistence and maintenance of HIV include macrophages, monocytes, dendritic cells, hematopoietic stem cells, microglial cells and astrocytes. On ART, the reservoir persists predominantly in a state of latency. However, incomplete virus suppression and reactivation can still occur, with some defective genomes producing viral proteins and replication competent genomes producing infectious virions that re-seed the reservoir. Created with BioRender.com

The latent reservoir is primarily found within resting memory CD4^+^ T cells, however macrophages, monocytes, dendritic cells, astrocytes, microglial cells and hemopoietic stem cells (HSCs) have also been implicated as contributing to latency and the persistence of HIV [105–107]. As such, reservoir sites have been found not only in circulation, but also in tissues, including lymph nodes, spleen, gut-associated lymphoid tissue, liver, lungs and immune privileged sanctuary sites, such as the brain and bone marrow, as well as others [105–107]. While there are several HIV cure strategies under investigation, they are all challenged by the heterogeneity of the latent reservoir, which is long-lived, self-replenishing, therapeutically refractory, widespread anatomically and immune evasive and thus it remains the major barrier to achieving an HIV cure (Fig. 3).

### Current HIV cure strategies

#### Hematopoietic stem cell transplant

The recipient of any HIV cure would ideally have no detectable RNA or DNA viral load, no viral transmission and no damage to their immune system in the absence of ART. HIV cure research focuses on two areas: elimination of proviral reservoirs or immunological control of proviral reservoirs, also known as a functional cure. Six people with HIV have been cured to date using stem cell transplantation [108–113]. The primary goal of the stem cell transplantation was to treat treatment resistant hematological malignancies, and used human leukocyte antigen (HLA) matched, non-relative donors. In five of the cases [108–112], donors with a rare homozygous mutation in *CCR5*, rendering cells inherently and highly resistant to HIV, were also used. While the stem cell transplantation cure approach has been successful for individuals achieving long-term remission, due to the high morbidity, mortality, expense and complexity of the procedure and its limited applicability and scalability, research efforts must be focused elsewhere.

#### Gene editing

RNA technologies for targeted genome editing are also of interest to HIV cure research, particularly the RNA-guided CRISPR-Cas9 system [114, 115]. CRISPR-Cas9 based gene therapies, have been used in vitro to disrupt HIV genes essential for viral replication [116] and human factors that are essential for viral entry such as *CCR5* and *CXCR4* [117, 118], however in vivo mouse model studies have focused on proviral DNA excision [119–121]. Recently, in vivo preclinical studies using a CRISPR-Cas9 construct, delivered intravenously via an adeno-associated viral vector, and designed to remove proviral DNA by targeting the 3’ and 5’LTR regions and Gag, demonstrated this approach to be safe and reduce proviral DNA in blood and tissues in non-human primate models of HIV by up to 95% compared to 20% in non-CRISPR animals [122, 123]. This has progressed into a Phase I, first in human study in people with HIV on ART, which is currently recruiting (NCT05144386, NCT05143307). Gene editing of *CCR5* in HSCs using zinc finger nucleases has also been demonstrated in vivo [124], with a Phase I clinical trial currently assessing the feasibility and safety of engraftment of these modified cells in people with HIV (NCT02500849). Other gene editing strategies for an HIV cure have been reviewed by Xun et al. [125].

#### Shock and kill

The ‘shock and kill’ strategy uses latency reversing agents (LRAs) to ‘shock’ latently infected cells to stimulate dormant HIV, to then ‘kill’ via susceptibility to HIV-induced cytopathic effect or immune-mediated clearance [126]. This elimination strategy uses LRAs simultaneously with ART to prevent *de novo* replication rounds and re-seeding of the reservoirs [126]. As the most extensively studied cure approach, many LRAs have been explored including epigenetic modifiers, such as HDAC and HMT inhibitors, or stimulators of transcription factors. HDAC inhibitors, such as vorinostat, panobinostat and romidepsin that promote chromatin relaxation and gene transcription, have been the most widely studied LRA in clinical research. While these drugs have demonstrated they can effectively activate latent HIV, they have failed to measurably reduce the size of proviral reservoirs or induce post-treatment control when used alone [127–130] or in combination with other therapeutics [131–133]. Recently, the anti-cancer therapy venetoclax has been shown to deplete HIV-infected CD4^+^ T cells from people with HIV on ART ex vivo and delay viral rebound following ART interruption in vivo in a humanized mouse model of HIV [134]. Further clinical investigation will be required to determine whether venetoclax is able to reduce the size of viral reservoirs in humans.

#### Block and lock

The key role epigenetic modulation plays in regulating HIV latency has inspired the ‘block and lock’ cure approach (reviewed in [135]). The premise of this strategy is to mimic natural HIV latency by inducing repressive post-translational epigenetic modifications to force HIV reservoir sites into a state of deep, and irreversible, latency. With the use of latency promoting agents (LPAs), viral transcription is ‘blocked’, and the promoter is ‘locked’ to permanently silence the provirus. This functional cure approach is not an unreasonable ambition, with a precedent set by 8% of the human genome being comprised of endogenous retroviruses, which do not produce infectious virus and are epigenetically regulated and maintained in a transcriptionally silent state [136, 137]. In addition, individuals that naturally control HIV to undetectable levels, either spontaneously (known as elite controllers) or following a transient period of ART (post-treatment controllers), provide further evidence that the goal of a functional cure is achievable [138, 139]. The use of siRNAs to epigenetically silence proviral HIV will be discussed in further detail in the section “Epigenetic silencing of HIV using RNAi”. Other LPAs explored to induce block and lock of HIV include small molecule inhibitors that inhibit the HIV trans-activator of transcription protein (essential for driving transcriptional elongation) [140–143] or host factors and signaling pathways required for viral expression [144–152] (summarized in Table 2).

**Table 2:** Summary of small molecule block and lock therapeutics

LPA name	Target	Mode of action	Type of studies	Ref
dCA	Virus	Tat inhibitor	In vivo	[140, 141]
Nullbasic	Virus	Tat inhibitor	In vivo	[142, 143]
ZL0580	Host	BRD4 inhibitor	In vitro, ex vivo	[144, 145]
CBL0100	Host	FACT complex inhibitor	In vitro	[146]
IM	Host	CDK9 inhibitor	In vivo	[147, 148]
pp242, Torin1	Host	mTOR inhibitor	In vitro, ex vivo	[149]
17-AAG	Host	HSP90 inhibitor	In vivo	[150]
GV1001	Host	HSP90 inhibitor	In vitro	[151]
LEDGIN	Host	LEDGF/p75 inhibitor	In vitro	[152]

Abbreviations: LPA, latency promoting agent; dCA, didehydro-cortistatin A; Tat, trans-activator of transcription protein; BRD4, bromodomain-containing protein 4; CBL0100, curaxin 100; FACT, facilitates chromatin transcription; IM, indirubin 3’-monoxime; CDK9, cyclin-dependent kinase 9; mTOR, mammalian target of rapamycin; HSP90, heat-shock protein 90; LEDGF/p75, Lens epithelium-derived growth factor; LEDGIN, LEDGF/p75 inhibitors

### Epigenetic silencing of HIV using RNAi

Due to its high mutation rate, treatment of HIV using therapeutic siRNA is complicated by the resulting extraordinary genetic diversity [153]. HIV type 1 (HIV-1), which accounts for 95% of cases globally, is classified into four groups (M, N, O, P), with group M being further divided into nine distinct subtypes (A, B, C, D, F, G, H, J, K) [154]. Several of these are further categorized into sub-subtypes and HIV is prone to recombination to produce circulating recombinant forms (CRFs).

Following the discovery of RNAi, siRNAs that harness PTGS were developed to inhibit key viral transcripts essential for replication, which showed initial success in vitro [155–157]. However, as PTGS is a transient process, requiring transcription to generate the target mRNA target, the rapid generation of escape mutants with point mutations, that abolished siRNA efficacy due to loss of the target sequence, or changes to RNA secondary structures was observed [158–160]. Designing siRNAs to target highly conserved viral regions and using a combination of siRNAs has been proposed as a possible solution. However, treatment with such a drug would still require regular life-long dosing, likely via the intravenous or subcutaneous route as seen with current approved RNAi therapeutics [25–30], which is substantially less practical compared to a single pill dose of ART.

The 5’LTR viral protomer offers a promising alternative target for therapeutic siRNA through the TGS RNAi pathway, which provides more durable silencing compared to PTGS. In addition, using a multiplex of siRNAs would achieve maximum sequence coverage of all circulating strains of HIV, such that one siRNA cocktail could treat most, if not all, cases globally. In addition, a combination of siRNAs would likely be robust against escape mutants in the event of low-level transcription. Surprisingly, the use of siRNAs to epigenetically silence HIV has gained little attention over the years, with few research groups focusing efforts into developing this method of functional cure (summarized in Table 3). Reasons for this are likely due to the poorer understanding of the TGS RNAi pathway and how it can be exploited therapeutically. Further, the difficulties faced with RNA delivery generally, compounded with the resting CD4^+^ T cells that are relatively refractory to traditional gene delivery approaches is a further major challenge to developing this functional cure.

The first siRNA therapeutic to epigenetically silence HIV was reported by our group in 2005 [161]. Termed siPromA, this therapeutic targets tandem NF-κB binding motifs within the 5’LTR viral promoter and was reported to induce potent viral suppression of up to 1000-fold for over 30 days, without off target-effects in vitro [40, 161–163]. These effects were accompanied by repressive epigenetic profiles associated with transcriptional silencing, including methylation of H3K9 and H3K27. In vitro studies using lentiviral vectors to constitutively express siPromA, demonstrated that HIV replication could be suppressed for over a year [164] and stably siPromA expressing cells were resistant to reactivation stimuli [165]. The researchers also discovered novel si143, targeting the 5’LTR upstream of siPromA and demonstrated that multiplexing these therapeutics could have an equivalent silencing effect compared to their single counterparts [162, 165]. The siRNA therapeutic approach was translated to mouse models in vivo, where mice were transplanted with human peripheral blood mononuclear cells (PBMCs) [166] or CD34^+^ HSCs [167, 168] transduced to stably express siPromA. In both studies, a significant reduction in intracellular HIV genomic RNA expression was observed in gene-modified mice expressing siPromA compared with controls, accompanied with an increased protection of CD4^+^ T cells from virus-induced death [166–168]. Recently, the first ever demonstration of nanocarrier facilitated nuclear delivery of siRNA, to induce TGS, was published using siPromA and a layer-by-layer nanoparticle [169]. This nanoparticle is multilayered and comprised of alternating assembly of of poly-L-arginine, a cationic polypeptide used for intracellular translocation of various cargoes, and biocompatible poly-4-styrenesulfonate. Although this particle was shown to deliver functional siRNA, its large size (~ 900 nm in diameter) is unsuitable for HIV as it would not be readily internalised by cells of the reservoir, such as T cells, or be able to enter sancturary sites, such as lymph nodes or brain [169].

Other 5’LTR targeted siRNA that induce epigenetic silencing of HIV have been reported. The siRNA termed LTR-362, which also targets the NF-κB binding motif within the HIV promoter, was identified by the Morris laboratory in 2006 [16]. Utilizing only the antisense strand, LTR-362 was found to inhibit HIV replication in vitro, which correlated with an increase in repressive epigenetic marks in cell lines [170]. To a lesser extent, silencing was also observed in human CD4^+^ T cells, where therapeutic efficacy was conferred up to a threshold where viral burden became overwhelming [171]. A subsequent study found that an siRNA-aptamer conjugate could successfully deliver functional LTR-362 in vitro, suppressing HIV infection approximately 10-fold [172]. When assessed in mouse models in vivo, the LTR-362-aptamer conjugate successfully repressed viral RNA levels in serum, however this was in the absence of CpG methylation, suggesting it was acting upon the flanking identical 3’LTR and in a PTGS fashion instead [172].

Singh et al. found in vitro treatment with the siRNA named S4 was able to suppress HIV subtype C production by up to 80% for 18 days post-infection in cell lines, and by almost 90% in primary human PBMCs for 24 days post-infection [173]. This silencing effect was associated with epigenetic marks associated with heterochromatization of the target loci [173]. However, S4 was intentionally designed to target the NF-κB motif of the HIV Subtype C promoter, the region of which is not conserved among other subtypes.

Developing therapeutic siRNAs for HIV presents many additional challenges than that of SARS-CoV-2, namely the requirement of long-term silencing coupled with significantly more complex target sites of disease. While lentiviral vector shRNA delivery is certainly advantageous by achieving constitutive siRNA production, and thus long-term silencing, this approach would require ex vivo cell modification and stem cell transplantation, hampering its scalability to all people with HIV. Further studies with a focus on developing improved in vivo delivery vectors, such as LNPs, that specifically target latent reservoir cells will be crucial to advancing HIV targeted siRNAs towards clinical trials.

As with SARS-CoV-2, a siRNA multiplex strategy will be critical to developing a broad-spectrum therapeutic. A multiplex approach has only been explored briefly by a few studies [162, 165], and no study has demonstrated multi-subtype efficacy of siRNAs against HIV. This is particularly important for a virus as genetically diverse as HIV, where there is an increased likelihood of at least one mismatch occurring, even when siRNAs have been designed to conserved regions of the 5’LTR. Indeed, prolonged silencing of HIV Subtype C primary isolate was observed for at least 21 days following treatment with S4 in PMBCs in the presence of a mismatch [173]. However, this same siRNA was shown to have no therapeutic effect against Subtype B when assessed. Other studies showed that one or more mismatches abolish effectivity completely or reduces silencing durability [162, 163]. These findings highlight the value of utilizing a multiplex of siRNAs to ensure the possibility of global impact. Detailed studies also exploring mismatch tolerance of TGS-inducing siRNAs and how this may influence the formation and maintenance of heterochromatin may facilitate future rational design of siRNAs against HIV.

It is also possible that a combination of cure strategies beyond siRNAs may also be required. Indeed, the sequential application of a shock and kill approach to reactivate accessible provirus, followed by the induction of block and lock to push remaining reservoirs further into latency has been proposed previously [174], however further research into blending approaches will be necessary.

**Table 3:** Summary of preclinical HIV targeted siRNA studies

siRNA name	5’LTR target site	Type of studies	Delivery vehicle	Ref
siPromA	NF-κB	In vivo	Lentiviral vector	[40, 161–168]
si143	AP-1/COUP-TF	In vitro	Lentiviral vector	[162, 165]
siPromA	NF-κB	In vitro	LBL nanoparticle	[169]
LTR-362	NF-κB	In vivo	A-1 aptamer	[16, 170–172]
S4	NF-κB (C only)	In vitro	Oligofectamine	[173]

Abbreviations: LTR, long terminal repeat; NF-κB, nuclear factor kappa B; AP-1, activator protein-1; COUP-TF, chicken ovalbumin upstream promoter-transcription factor; LBL, layer-by-layer

## Nanoparticle-based siRNA delivery systems in the clinic

**Fig. 4:**
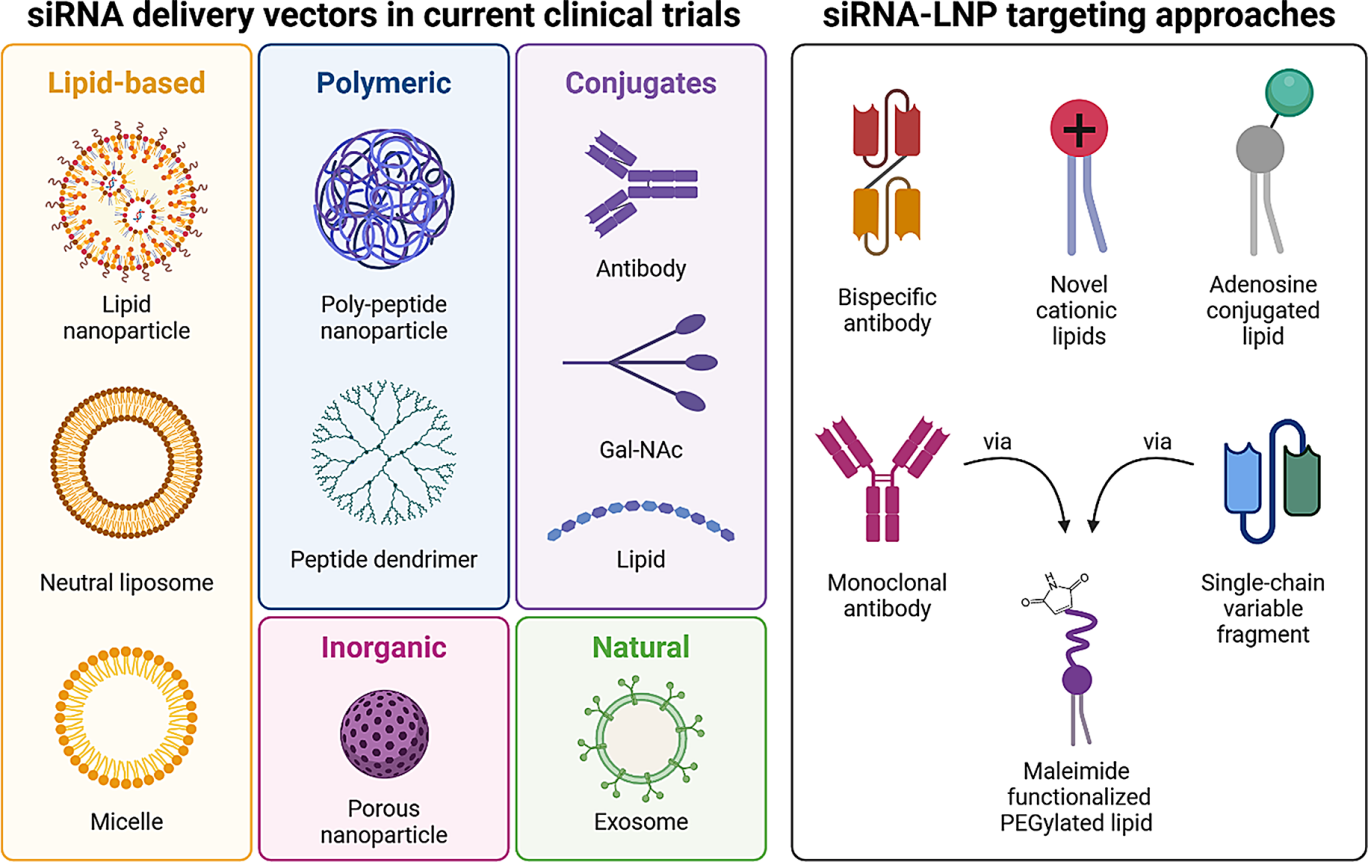
siRNA delivery vehicles and targeting strategies. Left: siRNA delivery vehicle classes in current clinical trials. Right: Surface functionalization strategies highlighted in this review in preclinical research for siRNA-LNP targeting. Created with BioRender.com

The intrinsic properties of siRNA presents several disadvantages that hinder its clinical success as a standalone therapeutic, including their rapid degradation in peripheral blood, and a uniform negative charge that renders siRNA unable to cross cellular membranes. To overcome these barriers, several delivery platforms have been developed for encapsulation and in vivo delivery of siRNA, with GalNAc and LNPs at the forefront of delivery technologies [25–30] (Fig. 4). These offer key advantages such as high entrapment efficiency of siRNA, high stability in blood circulation, low toxicity, facile penetration into the cell, and efficient endocytic trafficking that allow siRNA to eventually reach the cytoplasm, transfect the target cell and execute its silencing effect [175].

Of note, of the six FDA approved RNAi therapies, five utilize a GalNAc conjugate as an siRNA vector for cellular delivery [26–30]. GalNAc recognizes and attaches to the asialoglycoprotein receptors abundantly expressed on the surface of hepatocytes, promoting receptor-mediated endocytosis (RME) [176]. Despite being PTGS-harnessing therapies, each demonstrate remarkable silencing durability, with subcutaneous injection only required monthly (Givosiran, Nedosiran) [26, 30], every 3 months (Lumasiran, Vutrisiran) [27, 29] or every 6 months (Inclisiran) [28] after initial dosing. Mechanistic studies have shown this is owed to the stability of the chemically modified siRNA, which results in prolonged survival in, and release from, subcellular compartments [177]. Patisiran, on the other hand, employs a specialized LNP vector for siRNA delivery to the liver [25]. The enhanced liver uptake of Patisiran is facilitated by the adsorption of specific serum components to the surface of the LNP following IV administration, forming a biomolecular coat known as a ‘corona’ [178]. Such components include Apolipoprotein E (ApoE) and opsonins, which promote LNP binding to LDL-receptors highly expressed on hepatocytes, and their recognition by resident phagocytic cells in the liver, respectively [179, 180]. However, Vutrisiran, the newer second-generation RNAi therapy for hereditary amyloidosis now utilize a GalNAc conjugate, with reduced (every 3 weeks versus every 3 months) and less-invasive (subcutaneous versus intravenous) dosing requirements than that of Patisiran [29]. In addition to GalNAc and LNPs, other nanocarriers for siRNA delivery currently explored in clinical trials include polymeric [78, 181], inorganic [182] and lipid-based nanoparticles [183–185], exosomes [186] and conjugates [46, 187, 188] (Fig. 4, **left**). These systems are being developed to treat a range of diseases including viral infections, cancers and genetic disorders, with target sites in the liver, lung, pancreas, colon, skin and central nervous system (CNS) (summarized in Table 4). However, it is important to note that for a nanocarrier to be successfully translated into human use in the clinic, the manufacturing process must be scalable.

### Tools to modulate LNP biodistribution

The successful clinical translation of one LNP-siRNA therapeutic [25] and two LNP-mRNA vaccines [3, 4] has established that LNPs and RNA are a safe, efficacious and scalable technology platform. Coupled with the increasing availability of user-friendly LNP assembly platforms, such as NanoAssemblr^®^ microfluidic technology, which can rapidly generate high quality and reproducible particles [189], preclinical research into developing and optimizing LNP formulations has skyrocketed.

LNPs are the most advanced platform for delivering nucleic acids and are comprised of multicomponent lipid formulations containing four components; (i) an ionizable cationic lipid, (ii) a phospholipid, (iii) cholesterol and (iv) a polyethylene glycol (PEG)-conjugated lipid combined at a molar ratio of approximately 50:10:38.5:1.5 [190, 191]. Ionizable cationic lipids are critical for siRNA entrapment and endosomal escape, cholesterol and phospholipids are important for stability and biocompatibility and PEGylated lipids prevent LNP aggregation and are crucial for avoiding immune recognition and increasing the half-life in circulation which facilitates wider particle dissemination [190, 191].

However, despite these advances in nanomedicine, the accumulation and sequestering of nanoparticles from the bloodstream by the liver, spleen and kidneys following administration presents a significant obstacle [179, 192]. This interaction commonly results in reduced delivery to the target site, immune activation and increased hepatic toxicity [179, 192], and can severely hamper the effectivity of siRNA-nanoparticle systems towards infectious diseases, such as SARS-CoV-2 or HIV.

Redirecting nanoparticle biodistribution thus remains a challenge and requires changes to one or more nanoparticle properties including size, shape, composition, or surface engineering. A nanoparticle can be functionalized by decorating its surface with targeting ligands such as antibodies, peptides, or aptamers [193] (Fig. 4, right). Such targeting molecules are commonly conjugated covalently [193] and are used to induce uptake by a target cell via binding to specific cell-surface receptors or antigens [176]. Newer facile, non-covalent functionalization approaches have also been developed to form targeted nanoparticle systems [194–197]. These include bispecific antibodies that bind to PEG-coated nanoparticles in a single-step complexation. With simultaneous specificity for a target cell surface receptor, bispecific antibodies have been used to demonstrate targeted and enhanced delivery of chemotherapy [194, 195] and siRNA [196] in high-risk cancer models in vivo. Another self-assembly technology utilizes LNPs coated with an antibody crystallizable fragment (Fc) domain reactive lipoprotein to generate a flexible and customizable platform for mAb attachment and targeting, shown to efficiently redirect uptake of siRNA to different leukocyte subsets through mAb switching [197].

**Table 4:** Nanoparticle-based siRNA delivery platforms in current clinical trials

Delivery vector	Nanocarrier class	Disease	Target organ	Administration route	Ref	Clinical trial ID
Peptide-dendrimer	Polymeric	COVID-19	Lung	Inhalation	[78]	NCT05783206
Poly-peptide nanoparticle	Polymeric	Skin fibrosis	Skin	Intradermal	[181]	NCT04844840
		Skin cancers		Intratumorally		NCT05421013 NCT04669808
Porous nanoparticle	Inorganic	Skin fibrosis	Skin	Subcutaneous	[182]	NCT04707131
LNP	Lipid-based	Cancer	Lung, pancreas and colon	Intravenous	[183]	NCT03819387
Micelle	Lipid-based	Pulmonary fibrosis	Lung	Intravenous	[184]	NCT05984992
Neutral liposome	Lipid-based	Cancer	Not specified	Intravenous	[185]	NCT01591356
Exosome	Natural	Cancer	Not specified	Intravenous	[186]	NCT03608631
GalNAc	Conjugate	Chronic Hepatitis B	Liver	Subcutaneous	[46]	NCT04225715
Lipid conjugate	Conjugate	Early-onset Alzheimer’s Disease	CNS	Intrathecal	[187]	NCT05231785
Antibody conjugate	Conjugate	Muscular Dystrophy	Skeletal muscle	Intravenous	[188]	NCT05747924

Other approaches include the inclusion of specific lipids or identifying formulations to modulate organ biodistribution of nanoparticles. Cheng et al. demonstrated that supplementing Patisiran-based LNPs with a 5th lipid can drive differential LNP biodistribution in mice (an approach termed as selective organ-targeting or SORT), modulating LNP accumulation and delivery of mRNA or CRISPR/Cas9 therapy to the liver, spleen, or lungs, depending on the chemical structure of the additional lipid [198, 199]. The observed altered biodistribution profile was further identified to be specific to the formation of unique coronas of plasma proteins on the LNPs [200]. However, there is a lack of research studying these technologies in the delivery of RNAi for viral diseases, and further research is required to elucidate the mechanisms underlying corona formation, and how specific corona components interact with different tissues, organs, and cells.

#### Targeted LNP lung delivery for a COVID-19 RNAi therapy

The administration route has also been identified as playing a major role on the biodistribution of LNPs. For example, LNPs administered by the intramuscular and intraperitoneal route have been shown to have preferential nanoparticle accumulation in lymphoid tissues [201, 202], whereas intravenous administration sees predominant uptake in the liver, spleen and kidneys [179, 192, 203]. For an acute disease, such as COVID-19, where the respiratory route is the dominant site of infection [204], aerosolized delivery to the respiratory tract (upper and/or lower) is an appealing strategy. Indeed, all clinical trials assessing SARS-CoV-2 siRNA investigate administering each drug via inhalation (NCT05677893, NCT05941793, NCT05783206) [78]. Localized lower respiratory tract lung delivery via inhalation, or upper respiratory tract delivery via intranasal administration, offers many advantages including rapid absorption, lower dosing requirements, limited side effects and is non-invasive [205]. While localized administration mitigates the need for surface functionalization, several approaches may still be employed to maximize therapeutic efficacy. Zhang et al. reported increased siRNA silencing to airway epithelial cells in vivo mouse models of asthma through inhalation and targeting of siRNA-loaded LNPs to intracellular adhesion molecule-1 (ICAM-1) receptors [206]. ICAM-1 receptor has also been found to be elevated in lung tissue and plasma from critically ill COVID-19 patients, representing a promising receptor target for site-specific delivery of an RNAi therapy [207]. Other approaches exploited to improve LNP delivery to the lungs have included increasing the molar concentration of PEG to enhance mucus penetration when administered via inhalation [208] and adapting Cheng et al.’s selective organ-targeting LNP-mRNA approach [198, 199] for RNAi targeted lung delivery against SARS-CoV-2 [73, 74].

#### Parameters to consider for targeted delivery of an RNAi HIV cure

For a chronic disease, such as HIV infection, which has diverse and complex viral sanctuary sites [105–107], multi-targeting of siRNA nanoparticle systems to the diverse affected tissues may be a preferable strategy to follow, albeit complicated, to ensure widespread RNAi therapy dissemination and measurable therapeutic effects. Targeting LNP vectors to the CD4 receptor on T cells has been investigated in previous in vivo mouse studies, having shown to induce effective delivery of siRNA in CD4^+^ T cells circulating in peripheral blood, as well as those residing in spleen, bone marrow, and lymph nodes [209]. Similar T cell-targeting strategies using CD4 as an anchoring receptor have also been used to achieve mRNA delivery and translation in T-cell enriched tissues including spleen and lymph nodes [210]. LNPs targeting to murine CD5 receptor was used in a separate study to deliver and express chimeric antigen receptor (CAR)-encoding mRNA in peripheral blood circulating T cells, leading to successful therapeutic outcomes in a murine model of heart disease [211]. Another study exploited LNP targeting to murine CD3, which improved the delivery and expression of mCherry-encoding mRNA by mouse circulating T cells. However, these events led to a transient activation of circulating and splenic T cells accompanied by complex T cell depletion and migration processes [212].

Targeting LNPs and RNA delivery to other reservoir sites such as macrophages and dendritic cells has also been demonstrated to be successful in vivo in mouse models. In an attempt to mitigate inflammation injuries in the lungs, Fei et al. investigated the targeting of miRNA 146a-loaded LNPs to alveolar macrophages using mannose [213], which binds to CD206 receptor expressed on the surface of most tissue macrophages [214]. The authors observed preferential uptake of miRNA 146a-LNPs by lung macrophages, inducing a significant decrease in lung inflammation and increased lung function compared with LNP vectors lacking mannose [213]. Other studies exploring RNA delivery to macrophages have included the use of LNPs integrated with phosphatidylserine (PS), which is known to engage PS receptors on the macrophage surface [215, 216]. These studies demonstrated that PS positively increased endocytic activity and expression of exogenously delivered mRNA by resident macrophages of secondary lymphoid organs such as the spleen and lymph nodes [215, 216]. Separately, Uemura et al. synthesized a novel cationic lipid and demonstrated that incorporating this into their LNP formulations drove preferential siRNA delivery to macrophages and dendritic cells [217]. Murine CD205 receptor targeting has been demonstrated as another strategy to facilitate siRNA delivery to dendritic cells [218]. Lastly, LNP-assisted in vivo delivery of therapeutic RNA to other complex reservoir sites including HSCs and cells of the CNS via LNP targeting have also been made possible through targeting CD117 on bone marrow-resident HSCs [219, 220], LNP functionalization with adenosine for astrocyte delivery of siRNA therapy [221], and novel cationic lipids that facilitated brain infiltration and siRNA delivery to glioblastoma [222, 223].

It is important to highlight that while these studies provide valuable insights into strategies that can potentially facilitate targeted delivery of RNAi therapy to latently infected cells, it is clear that an HIV cure will require precise nanoparticle engineering to develop a system capable of delivering a therapeutic dose to all target cells and reservoir sites. In addition, transfection of resting human memory CD4^+^ T cells (the primary HIV reservoir) using LNPs has not been published to date. Furthermore, research into identifying new reservoir specific biomarkers may be advantageous to develop new targeting systems [224].

## Future directions

While antiviral siRNAs present a potentially powerful, scalable and versatile tool for treating and curing viral diseases, particularly SARS-CoV-2 and HIV, there are many remaining hurdles to overcome in the RNAi field. Screening and identifying novel antiviral siRNAs and their respective delivery vectors is time consuming, and exists largely in preclinical research, with few therapeutic candidates progressing to clinical trials. Establishing methodologies that enable high-throughput identification of lead therapeutic candidates will be essential moving forward. The parallel ongoing development of clinically translatable LNP targeting approaches, and discovery of the next highly successful ‘GalNAc’ equivalent, will be a critical direction of the field, and will facilitate the progressing therapeutic siRNAs beyond liver-manifesting diseases.

## Concluding remarks

Decades of research into stabilizing RNA technologies and improving delivery efficacy, coupled with the first mRNA vaccines approved for clinical use, has seen the exponential growth of RNA- based approaches in preclinical and clinical development in recent years. The clinical history of many acute and chronic viral infections, such as SARS-CoV-2 and HIV among others, which have been (and will likely continue to be) significant global burdens, would be transformed by the development of antiviral RNAi therapeutics. It is clear that ongoing, multidisciplinary collaborations into optimizing siRNA-LNP formulations will be required to evolve these platforms further. Therefore, now is the time for the research community and industry partners to unite in accelerating basic science discoveries through to clinical translation, and harness the full potential of RNAi therapeutics.

## Data Availability

Not applicable.
